# Structure-Function Relations in Oxaloacetate Decarboxylase Complex. Fluorescence and Infrared Approaches to Monitor Oxomalonate and Na^+^ Binding Effect

**DOI:** 10.1371/journal.pone.0010935

**Published:** 2010-06-03

**Authors:** Thierry Granjon, Ofelia Maniti, Yolanda Auchli, Pius Dahinden, René Buchet, Olivier Marcillat, Peter Dimroth

**Affiliations:** 1 Institute of Microbiology, Eidgenössische Technische Hochschule, Zürich, Switzerland; 2 Institut de Chimie et Biochimie Moléculaires et Supramoléculaires, Unité Mixte de Recherche 5246, Université de Lyon, Université Lyon 1, Centre National de la Recherche Scientifique, Villeurbanne, France; Griffith University, Australia

## Abstract

**Background:**

Oxaloacetate decarboxylase (OAD) is a member of the Na^+^ transport decarboxylase enzyme family found exclusively in anaerobic bacteria. OAD of *Vibrio cholerae* catalyses a key step in citrate fermentation, converting the chemical energy of the decarboxylation reaction into an electrochemical gradient of Na^+^ ions across the membrane, which drives endergonic membrane reactions such as ATP synthesis, transport and motility. OAD is a membrane-bound enzyme composed of α, β and γ subunits. The α subunit contains the carboxyltransferase catalytic site.

**Methodology/Principal Findings:**

In this report, spectroscopic techniques were used to probe oxomalonate (a competitive inhibitor of OAD with respect to oxaloacetate) and Na^+^ effects on the enzyme tryptophan environment and on the secondary structure of the OAD complex, as well as the importance of each subunit in the catalytic mechanism. An intrinsic fluorescence approach, Red Edge Excitation Shift (REES), indicated that solvent molecule mobility in the vicinity of OAD tryptophans was more restricted in the presence of oxomalonate. It also demonstrated that, although the structure of OAD is sensitive to the presence of NaCl, oxomalonate was able to bind to the enzyme even in the absence of Na^+^. REES changes due to oxomalonate binding were also observed with the αγ and α subunits. Infrared spectra showed that OAD, αγ and α subunits have a main component band centered between 1655 and 1650 cm^−1^ characteristic of a high content of α helix structures. Addition of oxomalonate induced a shift of the amide-I band of OAD toward higher wavenumbers, interpreted as a slight decrease of β sheet structures and a concomitant increase of α helix structures. Oxomalonate binding to αγand α subunits also provoked secondary structure variations, but these effects were negligible compared to OAD complex.

**Conclusion:**

Oxomalonate binding affects the tryptophan environment of the carboxyltransferase subunit, whereas Na^+^ alters the tryptophan environment of the β subunit, consistent with the function of these subunits within the enzyme complex. Formation of a complex between OAD and its substrates elicits structural changes in the α-helical as well as β-strand secondary structure elements.

## Introduction

Oxaloacetate decarboxylase is a member of the sodium ion transport decarboxylase (NaT-DC) enzyme family which also includes methylmalonyl-CoA, malonate and glutaconyl-CoA decarboxylases. These enzymes are found exclusively in anaerobic bacteria where they play a role in energy conversion [1]–[4]. As an example, oxaloacetate decarboxylase of *Vibrio cholerae* catalyses a key step in the fermentation of citrate, converting the chemical energy of the decarboxylation reaction into an electrochemical gradient of Na^+^ ions across the membrane. The Na^+^ gradient drives endergonic membrane reactions such as ATP synthesis, solute transport and motility.

**Figure 1:**
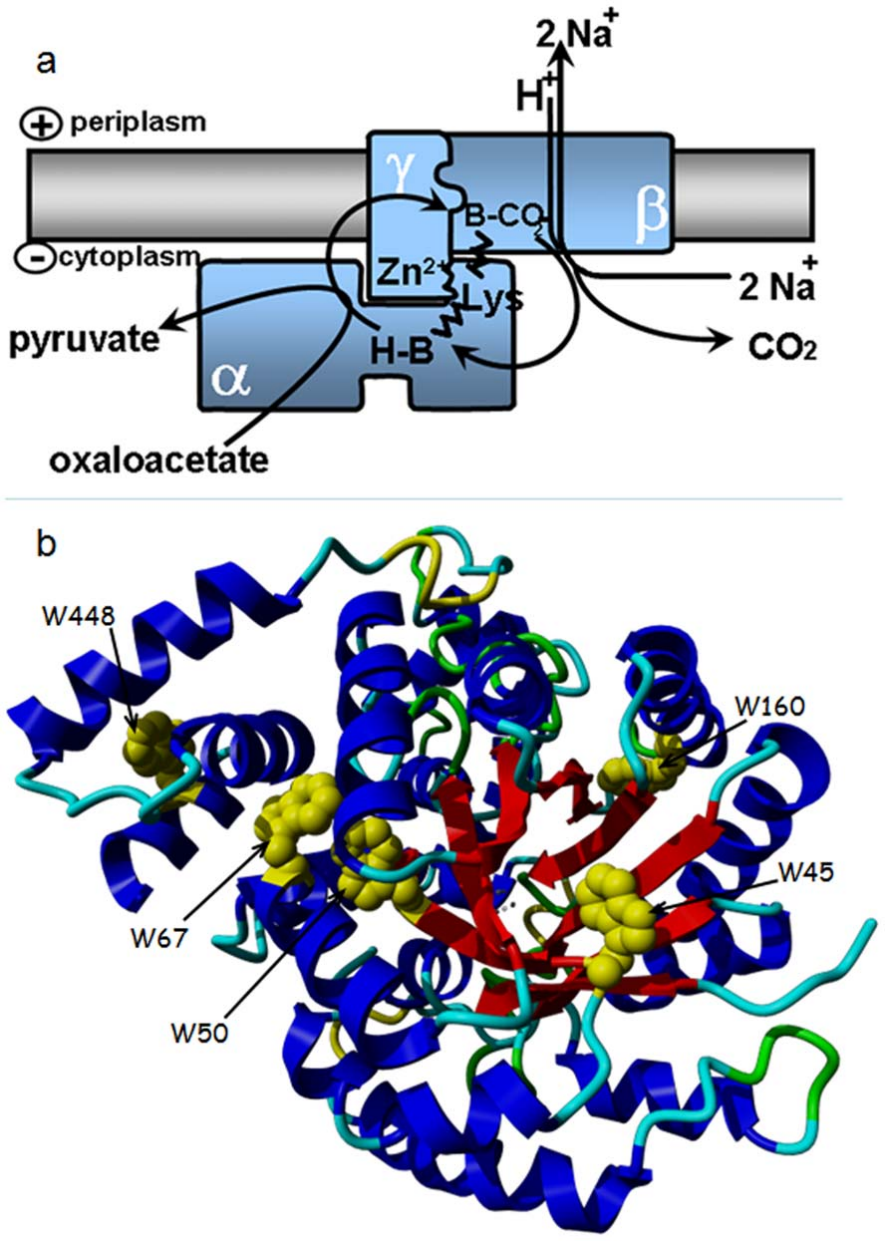
Organization of the OAD complex. **a. Structural model and catalytic events of the oxaloacetate decarboxylase.** Oxaloacetate decarboxylase is a membrane-bound enzyme complex composed of α, β, and γ subunits in a 1∶1∶1 molar ratio. The α subunit is soluble and harbors the carboxyltransferase catalytic site. The carboxyl group from position 4 of oxaloacetate is transferred to the biotin prosthetic group bound to the C-terminal biotin-binding domain. The carboxybiotin formed switches to the decarboxylase site on subunit β, where decarboxylation takes place and free biotin is regenerated, using one periplasmic proton. During the reaction, two sodium ions are translocated from the cytoplasm into the periplasm. Adapted from [9]. **b. Structure of the carboxyltransferase domain of OAD α subunit highlighting the position of tryptophan residues.** Four of them (positions 45, 50, 67, 160) are located within the catalytic α8β8 subdomain whereas a fifth tryptophan (W448) is located in the noncatalytic subdomain. This figure was drawn using YASARA (www.yasara.org) from PDB file 2NX9.

Oxaloacetate decarboxylase is a membrane-bound enzyme complex composed of α (OadA, 63–65 kDa), β (OadB, 40–45 kDa), and γ (OadG, 9–10 kDa) subunits in a 1∶1∶1 ratio (Fig. 1) [5], [6]. The α subunit is soluble and comprises three domains connected by a 40 amino acids long flexible linker peptide with a high content of proline and alanine residues [7]. The N-terminal domain of about 450 amino acid residues contains the carboxyltransferase catalytic site. The C-terminal domain of 70 amino acids contains the biotin-binding domain. The α subunit association domain is tightly bound to the C-terminal domain of the γ subunit, ensuring assembly and stability of the oxaloacetate decarboxylase complex [8]. Up to date, OAD structural knowledge is limited to the carboxyltransferase domain of the α subunit which shows a dimer of α_8_β_8_ barrels with an active site Zn^2+^ ion at the bottom of a deep cleft [9]. Based on this structure and on that of the related 5 S subunit of transcarboxylase from *P. shermanii* [10] and on additional biochemical experiments, it has been proposed that oxaloacetate binds to the Zn^2+^ containing site, and that the carboxyl group in position 4 is transferred to lysine 178 [9]. According to this scheme, pyruvate formed is subsequently replaced by the prosthetic biotin group which takes over the carboxyl group from the carbamylated lysine residue. A putative role of the γ subunit which also contains a Zn^2+^ ion and enhances the carboxyl transfer reaction is to stabilize the chemically labile carboxybiotin during its transfer from the carboxyltransferase site on subunit α to the decarboxylase site on subunit β. The β subunit is a very hydrophobic integral membrane protein that catalyses the decarboxylation of carboxybiotin coupled to Na^+^ ion transport across the membrane [11], [12]. The sequence of events taking place during oxaloacetate decarboxylation is depicted in Fig. 1a [13].

In this report, we used spectroscopic techniques to probe binding of two known OAD ligand: oxomalonate and Na^+^. Tryptophan fluorescence is one of the most widely used tools to probe tertiary structure fluctuations and dynamics of proteins. In order to evidence modifications that do not translate into variations of emission spectra, we used *Red Edge Excitation Shift* (REES) measurements, a phenomenon based on the electronic density redistribution under light absorption. REES refers to the increase in the emission wavelength occurring when the excitation wavelength is shifted towards the red edge of the absorption band. This increase arises from the slow rate of solvent relaxation around the excited-state fluorophore (tryptophan), which depends on the restriction imposed on the motion of solvent molecules in its immediate vicinity [14]–[16]. It does not provide information on the tryptophan itself, but rather on the organisation and the dynamics of the tryptophan environment. Thus, it becomes possible to monitor the environment-induced motional restriction imposed on the solvent molecules in the fluorophore vicinity.

Among OAD tryptophans, four residues (at position 45, 50, 67, 160) are all located near the surface of the catalytic α8β8 subdomain in the carboxyltransferase domain of the α subunit. A fifth tryptophan (W448) is located in the noncatalytic subdomain (Fig. 1b). Although none of these tryptophans is within the active site [8], some of them could be sensitive to dynamic modifications induced by ligand binding. No tryptophans are present in the γ subunit whereas the β subunit only contains one tryptophan in position 18.

Our findings show that oxomalonate binding affects tryptophan environment of the carboxyltransferase, whereas Na^+^ ions alter the tryptophan environment of the β subunit. These results are consistent with the function of these subunits within the enzyme complex. FTIR spectroscopy permitted us to monitor secondary structure modifications likely to occur after substrate binding [17], [18]. After oxomalonate binding, the OAD amide I absorption band components were shifted toward higher wavenumbers interpreted as a slight modification of the α helix over β sheet ratio.

## Results

### Tertiary structure changes caused by the binding of oxomalonate to OAD

**Figure 2:**
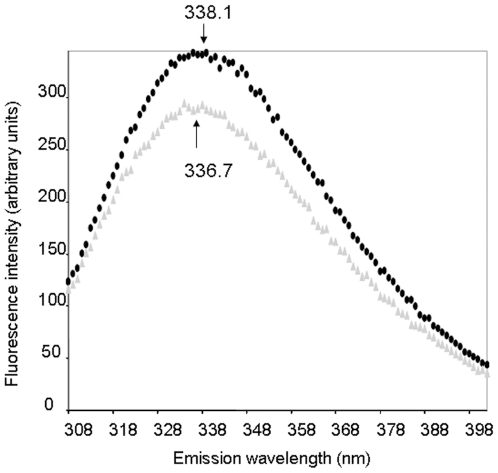
Fluorescence emission spectrum at 295 nm excitation wavelength of OAD in the absence (black circles) or presence (gray triangles) of 10 mM Oxomalonate. Each spectrum is the average of at least three determinations. Samples were in 250 mM NaCl, 0.5% Tween 20, 0.05% Brij 58 50 mM, Tris-HCl, pH 7.4 buffer.

Oxomalonate is a competitive inhibitor of oxaloacetate binding to the carboxyltransferase site on the OAD α subunit. To monitor structural changes elicited by inhibitor binding to this site, we measured fluorescence spectra of oxaloacetate decarboxylase and of its subunits in the absence or presence of the inhibitor. The oxaloacetate decarboxylase holoenzyme of *V. cholerae*, consisting of the three subunits α, β and γ in a 1∶1∶1 ratio, showed a typical tryptophan fluorescence spectrum with an emission maximum at 338.1 nm, when excited at 295 nm at 20°C (Fig. 2, black circles). In the presence of oxomalonate (Fig. 2, grey triangles), a shift of maximum emission wavelength, from 338.1 to 336.7 nm was observed, indicating a conformational change by which one or more of the tryptophan residues becomes less exposed to the aqueous environment. This result and the concomitant decrease of fluorescence emission intensity could also suggest involvement of some of the tryptophan residues in the interaction process. In order to obtain further information, based on the relaxation rate of tryptophan-surrounding solvent molecules and not on tryptophan itself, we also measured the dependence of the emission wavelength position upon excitation wavelength shift.

**Figure 3:**
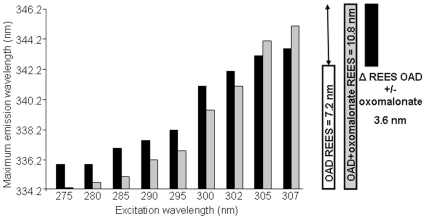
Red edge excitation shift observed as a result of oxomalonate binding to Oxaloacetate Decarboxylase. Maximum wavelength of the emission spectra at different excitation wavelengths of OAD in the absence (black) or presence (gray) of 10 mM Oxomalonate. Each point is the average of at least three determinations. White and gray bars (right) correspond to OAD REES observed in the absence and in the presence of oxomalonate respectively. Samples were in 250 mM NaCl, 0.5% Tween 20, 0.05% Brij 58, 50 mM Tris-HCl, pH 7.4 buffer.

Typical REES results are depicted in Fig. 3 and indicate a+7.2 nm shift in the absence of inhibitor (Fig. 3, black rectangles), from 335.8 to 343 nm, when changing the excitation wavelength from 275 to 307 nm. Such a REES indicates that fluorescence emission occurs before the dipolar relaxation due to restrictions of tryptophan-surrounding solvent molecule mobility in some areas of the protein.

After addition of 10 mM oxomalonate, the fluorescence maximum emission wavelength was blue-shifted by 1.6 nm when using a 275 nm excitation wavelength and red-shifted by 2 nm when using a 307 nm excitation wavelength with respect to OAD alone, leading thus to a 10.8 nm REES (Fig. 3, grey rectangles). This highlights modifications of the tryptophan-environment polarity and of tryptophan-surrounding solvent mobility. This effect of oxomalonate consisted in a decrease of solvent motion as expected in a non-fluid medium. Thus, ligand binding seemed to stiffen the tryptophan microenvironment, which is consistent with a change in the protein structure and more specifically a change of microenvironment in tryptophan residues.

### Effects of oxomalonate binding on the tertiary structure of the α subunit

**Figure 4:**
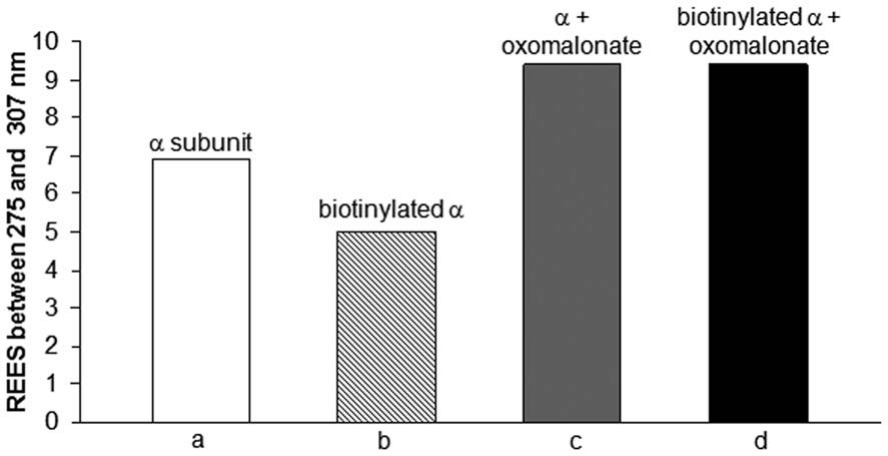
Effects of oxomalonate binding on the tertiary structure of OAD α subunit. REES variations observed on the nonbiotinylated (a, c) or biotinylated (b, d) α subunit in absence (a, b) or presence (c, d) of 10 mM oxomalonate. Each point is the average of at least three determinations. Samples were in 250 mM NaCl, 0.5% Tween 20, 0.05% Brij 58, 50 mM Tris-HCl, pH 7.4 buffer.

Spectroscopic measurements were also performed with OAD α subunit biotinylated or not (Fig. 4). The biotin-free α subunit and the biotin-containing α subunit showed a REES of +6.9 and +5 nm, respectively, indicating a restricted mobility of tryptophan surrounding solvent molecules in these proteins (Fig. 4, a and b). The 1.9 nm REES shift in the biotinylated vs. biotin-free protein reflects some release in the motional restrictions of the solvent molecules around tryptophan residues. OAD α subunit was able to bind oxomalonate as shown by the REES change from 6.9 nm to 9.4 nm (biotin-free) and from 5 nm to 9.4 nm (biotinylated) leading thus to a +2.5 nm or +4.4 nm REES shift, respectively (Fig. 4, c and d). Oxomalonate binding induced a similar REES effect on the whole OAD complex and on the α, or αγ subunits. We can then argue that the effect mainly concerned residues located in the α subunit (5 tryptophan residues, some of them located in the vicinity of the catalytic site) and to a lesser extent the single tryptophan residue of the beta subunit. The spectroscopic shifts observed upon oxomalonate binding to the oxaloacetate decarboxylase holoenzyme can thus largely be attributed to structural changes in the α subunit.

### Effects of oxomalonate binding on the tertiary structure of the α subunit and αγ complex of OAD

**Figure 5:**
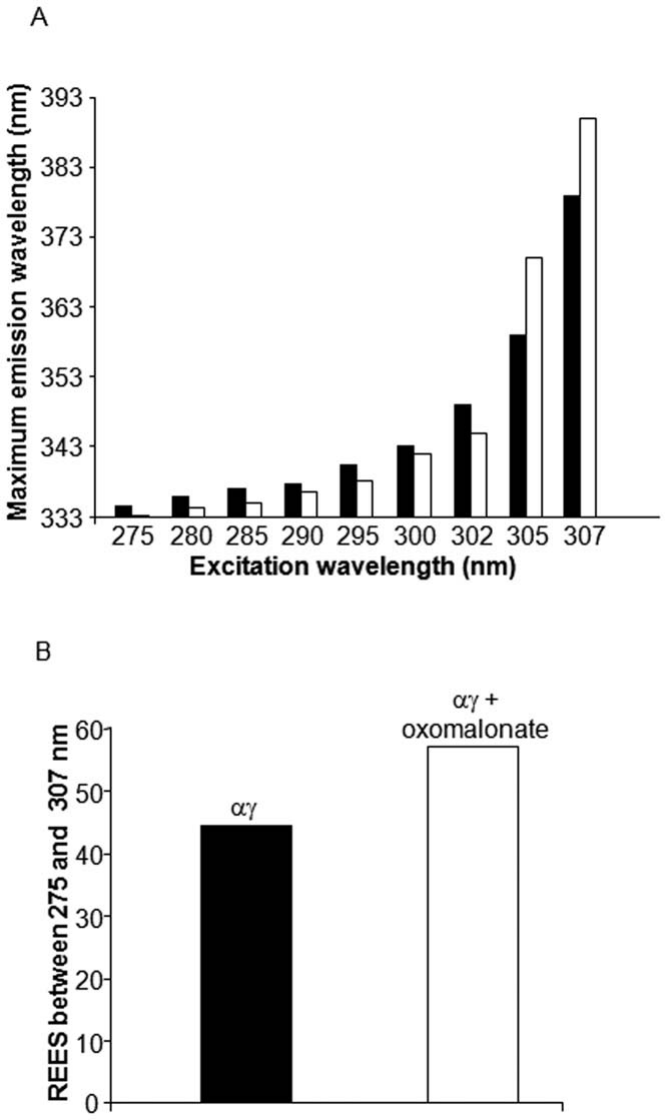
Red edge excitation shift observation as a result of the oxomalonate effect on the OAD αγ subunits structure. A. Maximum wavelength of the emission spectra of OAD α subunit at increasing excitation wavelengths in the absence (black rectangles) or presence (white rectangles) of 10 mM Oxomalonate. B. REES observed on the αγ subunit spectrum in the absence (black) or presence (white rectangles) of 10 mM Oxomalonate when the excitation was shifted from 275 nm to 307 nm. Each point is the average of at least three determinations. Samples were in 250 mM NaCl, 0.5% Tween 20, 0.05% Brij 58 50 mM, Tris-HCl, pH 7.4 buffer.

To understand the mechanism of substrate binding to OAD and the importance of each subunit in the catalytic mechanism, the effect of the γ subunit on the α subunit's fluorescence properties was determined. As the γ subunit does not contain tryptophan residues, any effect on the fluorescence spectrum is to be attributed to structural changes within the α subunit. The αγ complex exhibited a huge +44.4 nm REES (emission was shifted from 334 nm to 378.4 nm when excitation was shifted from 275 nm to 307 nm) (Fig. 5 a and b, black rectangles). Oxomalonate interacts with this complex and induced a further +12.4 nm shift of the REES (Fig. 5 a and b, white rectangles). These results are consistent with the notion that oxomalonate binding to the α subunit is not significantly influenced by the formation of complexes with the other subunits.

### Effects of Na+ ions on the tertiary structure and decarboxylase activity of OAD

**Figure 6:**
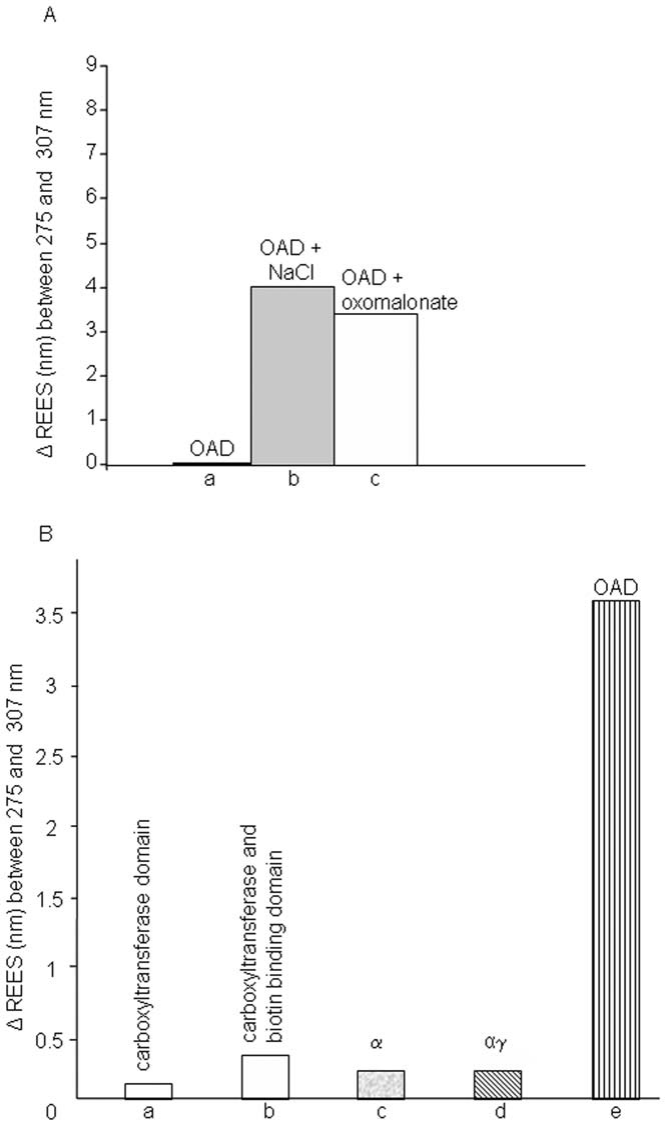
Influence of substrate binding on OAD and OAD subunits. A. OAD REES variations in the absence of substrates (a) or in the presence of either 250 mM NaCl (b) or 10 mM oxomalonate (c). OAD was purified in 250 mM KCl, pH 7.4, 0.5% Tween 20, 0.05% Brij 58, 100 mM KH_2_PO_4_ buffer. REES of OAD in KCl-containing buffer (i.e. 3.8 nm, not shown) was taken as reference to calculate the REES variation. B. Na^+^ (250 mM) influence on REES variation of carboxyltransferase domain of the α subunit (a), reconstituted carboxyltransferase and biotin binding domain of the α subunit (b), the whole α subunit (c), αγ complex (d), and the OAD complex (e).

The effects of NaCl on both the structure of the enzyme and its ability to interact with oxomalonate were investigated. For this purpose, OAD was purified using 250 mM KCl buffer instead of NaCl. Under those conditions, OAD exhibited a 3.8 nm REES (not shown). Addition of 250 mM NaCl to this sample induced a further 3.4 nm REES variation (Fig. 6A b). Of interest, although OAD was active in 250 mM potassium chloride buffer, the decarboxylase specific activity increased from 4.5 U/mg in KCl-containing buffer to 21 U/mg in the presence of NaCl.

Finally, oxomalonate binding to OAD purified in a KCl-containing buffer induced a 3 nm REES variation (Fig. 6A c). These findings indicated that OAD tertiary structure was sensitive to the presence of NaCl, although oxomalonate was still able to bind to the enzyme even in the absence of sodium ions.

### Effects of Na+ ions on the tertiary structure of α and αγ subunits of OAD

Low REES variations were recorded upon Na^+^ binding to the carboxyltransferase (Fig. 6B a) and biotin-binding domain of the α subunit (Fig. 6B b), the whole α subunit (Fig. 6B c) and αγ complex (Fig. 6B d) compared to the above described increase observed for the OAD complex (Fig. 6B e). This indicated that in the absence of the β subunit, sodium ions did not affect the mobility of tryptophan-surrounding solvent molecules.

### Secondary structure of OAD, αγ and α subunits

**Figure 7:**
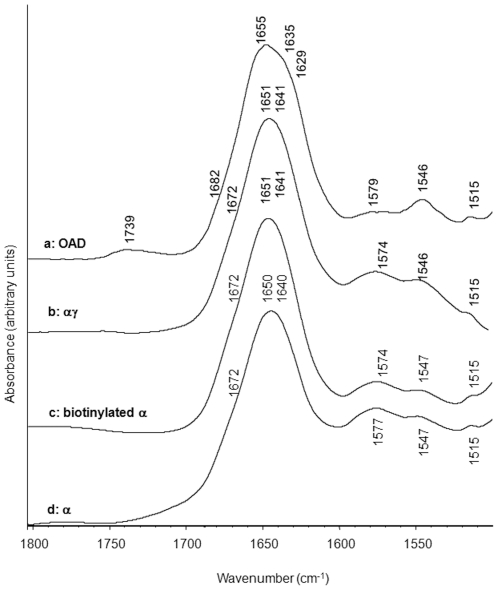
Amide I comparative secondary structure spectra of OAD and OAD subunits. OAD (a), αγ (b), biotinylated α (c), and nonbiotinylated α (d) subunits.

The spectra of OAD (Fig. 7a) and of αγ complex (Fig. 7b), as well as those of biotinylaled and non-biotinylated α subunit (Fig. 7c and 7d) exhibited a main component band around 1655—1650 cm^−1^, characteristic of a high proportion of α helix structures [19], [20]. However, the α helix band of the ΟΑD is centered at 1655 cm^−1^ (Fig. 7a) instead of 1651 cm^−1^ for the α and αγ subunits (Fig. 7b, 7c, and 7d). Indeed, fitting curves of the amide I band of α, with or without biotin, as well as αγ complex show main bands at 1650–1651 cm^−1^ characteristic of α helices (Figure S1). Minor component bands were located at ≈1635 cm^−1^, 1640–1641 cm^−1^ and 1685–1675 cm^−1^. This suggested the presence of random structures (1640 cm^−1^) and of a small amount of β sheet (1635 cm^−1^ and 1685–1675 cm^−1^) [7], [12] in the α subunit (Fig. 7c and 7d) and in the αγ complex (Fig. 7b).

In the case of the OAD complex, β sheet contribution becomes more important, with two shoulders at 1629 and 1635 cm^−1^ as determined using the second derivative method (not shown). Other bands, corresponding to COO^-^ vibrations of aspartate and glutamate (1587–1566 cm^−1^), and tyrosine residues (1515 cm^−1^) [24], are observed together with the amide-II band centered at 1546–1547 cm^−1^.

A small band around 1739 cm^−1^, related to ester carbonyl stretching mode, is visible on both α, αγand OAD infrared spectra (Fig. 7). This band was tentatively attributed to the ester group of Tween 20, remaining lipids from the purification process or protonated aspartic acid residues.

### Comparative secondary structural effects of oxomalonate binding to OAD, αγ and α subunit

**Figure 8:**
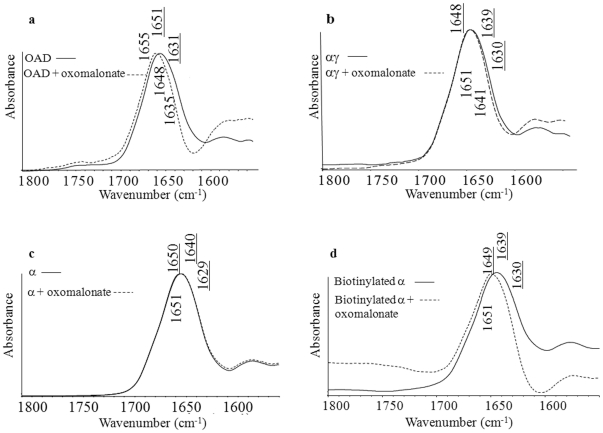
Effect of oxomalonate binding on OAD and OAD subunits secondary structure. (a) OAD in presence of NaCl, (b) αγ subunit, (c) α subunit, (d) biotinylated α subunit. Full and dotted lines represent the complexes without and with oxomalonate respectively.

Secondary structural effects of oxomalonate binding on OAD, αγ, or α with or without biotin were also assessed using FTIR spectroscopy. Our results revealed that, in the presence of oxomalonate, band components of OAD were shifted as compared with the individual protein spectrum (Fig. 8 a), namely from 1655–1651 cm^−1^ to 1653–1648 cm^−1^ for α helices and from 1631 to 1635 cm^−1^ for β sheets.

In addition, other subtle changes can be seen in the 1648 cm^−1^ and around 1700 cm^−1^ region, which can be associated with slight variations in the loop and β sheet structures of the protein.

Binding of oxomalonate to the αγ complex induced a shift in the amide I band vibrations from 1648 to 1651 cm^−1^, as well as a small change from 1639 to 1641 cm^−1^ (Fig. 8b). Changes also occurred in the amide II region with shifts of band vibrations from 1550 to 1546 cm^−1^ (Fig. 8) and from 1516 to 1514 cm^−1^(not shown).

Minor modifications also occurred in the COO^-^ vibrations of Aspartate and Glutamate, with a shift from 1580 to 1577 cm^−1^ (Fig. 8).

Concerning the α subunit, no major difference can be observed in the spectra obtained either in the presence or absence of oxomalonate (Fig. 8c). However, when the α subunit was biotinylated, spectra presented almost exactly the same differences than those observed with the OAD complex, indicating that, after oxomalonate binding, the biotinylated α subunit underwent smaller, but similar structural changes to those observed on the OAD (Fig. 8d). Indeed α helix and random structure-corresponding vibrations respectively shifted from 1649 cm^−1^ and 1638 cm^−1^ in the absence of oxomalonate to 1652 cm^−1^ and 1644 cm^−1^ in its presence. Hence, formation of complexes between the protein and its substrate appear to generate structural changes in the α-helical as well as β-strand secondary structural elements.

### Effects of Na+ ions on the secondary structure of the OAD complex

**Figure 9:**
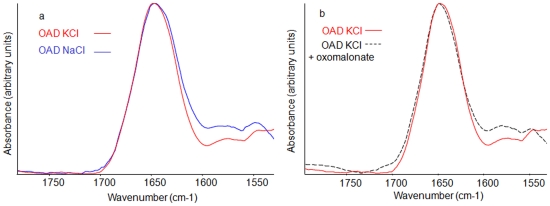
Na+ influence on OAD secondary structure and on the ability to bind oxomalonate. a. OAD secondary structure in the absence of NaCl (red line) and after the addition of 250 mM NaCl (blue line). b. OAD secondary structure in the absence NaCl before (red full line) and after addition of 10 µM oxomalonate (black dotted line). OAD was purified in 250 mM KCl, pH 7.4, 0.5% Tween 20, 0.05% Brij 58, 100 mM KH_2_PO_4_ buffer.

The IR spectrum of OAD in the absence of Na^+^ (OAD purified in KCl 250 mM buffer) showed main bands at 1653 cm^−1^, corresponding to α helix vibration, as well as at 1645 cm^−1^, related to random structures (Fig. 9a, red line). After addition of Na^+^, the α helix vibration band was shifted to a higher wavenumber, 1655 cm^−1^, and bands corresponding to β sheets appeared at 1682, 1635 and 1629 cm^−1^ (Fig. 9a, blue line). These values corresponded to the ones determined for the protein purified in the presence of Na^+^ (Fig. 8a). The 1579 cm^−1^-COO^-^ vibration band, observed in presence of Na^+^ ions, was shifted to 1576 cm^−1^ in the absence of Na^+^. The effect of oxomalonate in the absence of NaCl was less important than the effect observed in the presence of NaCl (Fig. 9b).

## Discussion

### Tertiary structure of NaT oxaloacetate decarboxylase: effect of cation and/or substrate binding

Among various fluorescence techniques, fluorescence quenching, resonance energy transfer, and polarization measurements yield information about the fluorophore itself, while REES provides information about the relative rates of solvent (water in biological systems) relaxation dynamics, which is not possible to obtain by other techniques. Since the dynamics of hydration are directly associated with the functionality of proteins, REES is a sensitive tool to explore the organization and dynamics of soluble and membrane proteins.

It is usually assumed that fluorescence emission occurs, after dipolar relaxation, from a relaxed state and thus that the emission wavelength is independent from the excitation (Kasha rule) [21]. However, in a more viscous medium, aromatic fluorophore fluorescence spectra can depend on the excitation wavelength [22]. Such an excitation wavelength dependency of emission spectra was specifically found with proteins exhibiting a fluorescence maximum between 325 and 341 nm [23]–[25]. Due to solvent motional restriction, the fluorescence life-time of molecules is shorter than solvent relaxation life-time. Thus, emission of fluorescence directly occurs from the excited state. The shift of the excitation wavelength to the red edge and far anti-Stokes region of the absorption spectra induces a red shift of the maximum emission wavelength of the fluorophore, leading to the so-called Red Edge Excitation Shift effect (REES). (For in-depth review see reference [22]).

In this present work, the REES exhibited by the OAD (around 7 nm), in the absence of any substrate is consistent with an interfacial localization of tryptophan residues, exhibiting slow solvent relaxation processes [26]. These interfacial regions are known to participate in charge-charge, as well as in hydrogen bonded, intermolecular interactions [27].

The higher REES observed when OAD was in the presence of oxomalonate (+3.6 nm) indicated that the average microenvironment of tryptophan residues became more ordered, showing an interaction between those compounds, as well as structural modifications (Fig. 3). Moreover, some of the enzyme tryptophan residues were directly affected by oxomalonate binding, as shown by both the 2 nm blue shift of the emission wavelength from 338 to 336 with 295 nm excitation wavelength and the clear fluorescence intensity quenching (Fig. 2). Those changes could also correspond to a minor modification of tryptophan “embedding” in the new conformation.

The carboxyltransferase domain contains four tryptophan residues (at position 45, 50, 67, 160) which are all located near the surface of the catalytic α8β8 subdomain and could therefore be responsible for the signal shift. The fifth tryptophan (W448) which is located in the noncatalytic subdomain is thus less likely to contribute to the fluorescence quenching by the inhibitor binding (Fig. 1b).

Taking these structural data into account, our spectroscopic results suggest that oxomalonate elicits global structural changes within the carboxyltransferase affecting the dipolar relaxation rate change of solvent molecules surrounding the whole tryptophan population and thus mobility of those solvent molecules at the surface of the protein.

The 7.2-nm (Fig. 3) and 3.4-nm REES (Fig. 6) observed for OAD in NaCl and in KCl buffer, respectively, attributed to the relaxation of the protein-water dielectric environment of the fluorophore residues, indicated that the average environment of the tryptophan residues in the OAD is less dynamic in the presence of sodium ions than in the presence of potassium ions. These observations are consistent with a specific effect of sodium ions on the β subunit and a direct implication of this subunit in the sodium translocation process [11]. Indeed, sodium replacement by potassium only affected the β subunit of the OAD complex as a REES effect was observed with neither the α subunit nor the αγ complex (Fig. 6B) after cation-replacement.

Whatever the structural effect of Na^+^ ions, it did not affect oxomalonate binding to OAD, as shown by the 3 nm REES variation observed in Fig. 6Ac. Moreover, besides the fluidity change, decarboxylase specific activity of the enzyme increased 4 to 5 times in the presence of NaCl as compared with KCl. Thus, our findings indicated that REES changes are sensitive to the nature of univalent cations, suggesting that REES could indirectly probe the dynamics of the active site.

### Secondary structure of OAD and of the different OAD subunits association

As shown above, fluorescence measurements strongly suggest that the conformation of the enzyme subunits changes when assembled in the OAD complex, as well as upon substrate binding. The structure of each subunit and the effect of substrate binding to the protein complex were therefore investigated by means of FTIR spectroscopy.

The strong absorption in the 1660–1650 cm^−1^ interval range (amide I mode) and around 1540 cm^−1^ (amide II mode) displayed by the different enzyme complexes indicated that secondary structure of OAD, was dominated by high α helices content [28], [29]. A close analysis of OAD and OAD subunits spectra however reveals the presence of absorption bands at two distinct frequencies in the 1660–1650 cm^−1^ range: at 1651 cm^−1^ for α, α-biotin and αγ complex and around 1655 cm^−1^ for the OAD complex. This can either be due to a shift of α helix absorption towards higher wavenumbers or to the co-existence of two types of α helical structure. A fit of the amide I band of the OAD complex in agreement with the last hypothesis is presented in Figure S1. This result bears some similarity with the α-helical signal of bacteriorhodopsin, in which two major bands can be distinguished at 1669 and 1658 cm^−1^ [30], [31]. The coexistence of two α helical structures at 1658 and 1650 cm-1 was suggested in the secretory phospholipase A2 once bound to lipid bilayers [32]. The band at 1658 cm^−1^ could correspond to more flexible and more dynamic α helices than the 1650 cm^−1^ one. It is thus an attractive possibility that the IR absorption bands in the 1660–1650 cm^−1^ interval of the OAD may arise from different α-helical components coexisting in the structure of the functional enzyme.

Additional bands were present in the amide I region, although their interpretation is more complex. The additional band at 1646/1647 cm^−1^ could arise from either 3_10_ helices (i.e. type III β turns), open loops, or even strongly H-bonded α helices [29], [33], . The bands at 1638 and 1629 cm^−1^ can be considered as a mixture of β sheets, 3_10_ helices and open loops [29], [33].

Secondary structure spectra of the α subunit as well as the αγ subunit (Fig. 8) were also dominated by α helices. Interestingly, no drastic effect from the biotinylation process was observed on the α subunit secondary structure. In the same way, although the γ subunit significantly affects the tryptophan-surrounding solvent molecule mobility and viscosity, as shown by the huge 44.4 nm REES effect indicating perturbation of the overall tertiary structure of the α subunit (Fig. 5), no significant changes were observed between α and αγ subunit secondary structures (Fig. 7). Indeed, in the amide I region, α and αγ spectra are superimposed. A 1574–1579 cm^−1^ shift can be observed for the α and αγ spectra as compared to the OAD spectrum. OAD, α, and αγ subunits respectively contain 100, 75 and 78 groups susceptible to absorb in the 1580 cm^−1^ region. This shift could therefore be related to a change in the Asp/Glu ratio.

In the presence of the β subunit, a modification in the α helix/β sheet ratio was observed, with an increase in the α helix content and structural rearrangements affecting β sheets. This intermolecular β sheet formation could originate from structural modification imposed by the β subunit on the complex, leading to perturbation of α helices and formation of new β sheets. They could also reflect β sheet presence in the β subunit. Those β sheets could correspond to the undefined part of the predicted structure between helix III and IV [11], [35]. In the absence of Na^+^ ions, the β sheet absorption band in the 1629–1635 cm^−1^ region was less important (Fig. 9a). However, addition of Na^+^ to this sample induced an increase in the absorption band in this spectral region (Fig. 9b). As REES measurements indicated that Na^+^ binding mainly affected the β subunit, this result supports the hypothesis that the absorption bands at 1628 and 1631 cm^−1^ mainly reflect β sheet presence in the β subunit.

### Effect of oxomalonate binding on the OAD secondary structure

Effects of oxomalonate binding on OAD, αγ, or α secondary structure were also assessed using FTIR spectroscopy.

Results revealed the formation of a complex between the protein and its substrate which appears to generate structural changes in the α-helical as well as β-strand secondary structure elements. Indeed, in the presence of oxomalonate, the main band components of OAD were shifted compared with the individual protein spectrum (Fig. 8a). These changes resulted from an environmental alteration implying a decrease of hydrogen bonding strength within α helices and between β-strands [29].

Other spectral peaks suggest changes in the protonation state and/or environment of acidic residues as well as modification in the contribution of other side chain residues [36]. Changes in the Arg side chain may give rise to a peak at 1671 cm^−1^ [36]). Small peaks at 1622 and 1614 cm^−1^ might be a mixture of absorption bands of Asn, Gln, Trp, Tyr side chains and of β sheet components.

Some of these effects were less important with isolated OAD subunits. As shown in fig. 8b, although the observed shift is weaker than in the case of OAD, oxomalonate was able to bind to the αγ complex and provoke structural variations. Plots of α subunit spectra in the absence or presence of oxomalonate did not show significant differences (slight shift 1649–1651 cm^−1^) (Fig. 8c). However, when the α subunit was biotinylated, we observed the same features than with OAD, suggesting that most of the spectral structural effects observed upon oxomalonate binding to OAD did indeed occur within the α subunit (Fig. 8d).

## Materials and Methods

### Recombinant DNA techniques and sequencing

Extraction of plasmid DNA, restriction enzyme digestions, DNA ligations, and transformation of *E. coli* with plasmids were carried out by standard methods [37], [38]. PCRs were performed with an air thermo-cycler (Idaho Technology, model 1605) using *Pfu* polymerase. The oligonucleotides used were custom-synthesized by Microsynth (Balgach, Switzerland). All inserts derived from PCR as well as ligation sites were checked by DNA sequencing according to the dideoxynucleotide chain-termination method [39] by Microsynth (Balgach, Switzerland).

Construction of expression plasmids for the α subunit or for αγ subunit was done as described in [8].

### Strains and growth conditions

For general cloning purposes *Escherichia coli* DH5α (Bethesda Research Laboratories) was used. Recombinant proteins were synthesized in *E. coli* C43(DE3) [40] and *E. coli* BL21 Star(DE3) cells (Invitrogen). Cells were routinely grown in Luria Bertani medium (LB) supplemented with 10 g/l NaCl. To increase biotinylation efficiency, culture medium contained 10 µM (+)-D-biotin. Expression was induced with 100 µM IPTG and the cells were harvested after 3 to 4 h at 30°C and 180 rpm.

### Separation of cytosolic and membrane fraction

For the preparation of cell extracts, cells from expression cultures (1 g wet weight) were resuspended in 7 ml buffer containing 0.2 mM diisopropylfluorophosphate and 50 µg DNase I as described in (8). After three passages through a French pressure cell at 110 MPa intact cells and cell debris were removed by centrifugation (30 min at 13000× g). The cell-free supernatant was subjected to ultracentrifugation (1 h at 200000× g) to separate the cytosolic fraction and the membrane fraction.

### Purification of biotinylated α or αγ

The purification process was performed as previously described (8). Appropriate plasmids were expressed in *E. coli* C43(DE3). The cells were resuspended in buffer A (50 mM Tris-HCl, pH 8.0, 50 mM NaCl) containing 1 mM MgK_2_EDTA. The cytosolic fraction was prepared as described above and applied to a monomeric avidin-Sepharose column, which was washed with 8 bed volumes of buffer A. Biotinylated protein was eluted with 1 bed volume of buffer A containing 5 mM (+)-D-biotin.

### Purification of non-biotinylated α or αγ

Cells from expression cultures of *E. coli* C43(DE3) containing the appropriate plasmids were suspended in HisBind buffer (500 mM NaCl, 50 mM Tris-HCl, pH 8.0,) containing 1 mM MgK_2_EDTA. The cytosolic fraction was prepared as described above and applied to a Ni-NTA agarose column which was washed with 15 and 11 column volumes HisBind buffer containing 5 and 10 mM imidazole, respectively. Specifically bound protein was then eluted with 5 column volumes HisBind buffer containing 150 mM imidazole. The biotinylated fraction of α or αγ was removed by passing the eluate through an avidin-Sepharose column. The biotin-free protein running through the column was dialyzed overnight against 50 mM Tris-HCl, pH 8.0, and 50 mM NaCl at 4°C.

### Purification of OAD from V. cholerae cells

OAD was purified from *V. cholerae* cells grown anaerobically on citrate as previously described [41], [42]. Briefly, *V. cholerae* cells were disrupted by sonication and the cell lysate centrifuged (200 000× g, 1 h) to collect the membranes. Membranes were resuspended in 50 mM Tris-HCl 250 mM NaCl buffer, pH 8.0, and solubilized by treatment with 2% Triton X-100 for 10 min at 4°C. The clear supernatant obtained after centrifugation at 220000× g for 35 minutes was applied to a monomeric avidin-Sepharose column (5 ml bed volume), equilibrated with 50 mM Tris-HCl buffer, pH 8.0 containing 50 mM NaCl, 0.5% Tween 20 and 0.05% Brij58. Biotinylated proteins were eluted with 5 mM (+)-D-biotin.

### Concentration of purified protein

Protein was concentrated using a 2 mL anionic exchange column (Fractogel EMD TMAE 650 (S), Merck) equilibrated with 50 mM Tris-HCl buffer, pH 8.0 containing 50 mM NaCl (α or αγ) or with 50 mM Tris-HCl buffer, pH 8.0 containing 50 mM NaCl, 0.5% Tween 20 and 0.05% Brij58 (holoenzyme). Pure 200 µL protein fractions were eluted with 50 mM Tris-HCl buffer, pH 8.0 containing 250 mM NaCl, 0.5% Tween 20 and 0.05% Brij58. For experiments performed in the absence of Na^+^, NaCl was replaced by 250 mM KCl. Protein concentration was determined by the BCA protein assay (Pierce, Rockford, IL) using bovine serum albumin as standard. SDS–PAGE was performed as described [20]. Gels were stained with Coomassie Brilliant Blue R-250 or with silver [21].

### Enzyme assay

The decarboxylation activity was determined with the spectrophotometric assay at 265 nm as described [43].

### Fluorescence Measurements

The maximum emission wavelength, recorded between 306 and 550 nm, was measured at an excitation wavelength ranging from 275 to 307 nm with a RF-5001 PC Shimadzu spectrofluorimeter. The excitation and emission band-pass values were 1.5 and 3 nm respectively. Spectra were recorded at 20°C, using a 1 cm-path length thermostated quartz cell. All spectra were corrected by the baseline spectra of water Raman peak contribution. Every fluorescence spectrum was representative of at least three independent measurements.

*Infrared Spectroscopy*. Protein samples were prepared as previously described, but the last Fractogel chromatography step was done in a ^2^H_2_O buffer containing 50 mM Tris-HCl, p^2^H 7.4 and 50 mM NaCl. The p^2^H was measured with a glass electrode and was corrected by a value of 0.4 according to Glasoe and Long [44]. Protein samples in ^2^H_2_O buffer with or without oxomalonate were loaded between two CaF_2_ circular cells, with a 50 µm Teflon spacer. Infrared spectra were recorded with a Nicolet 510 M FTIR spectrometer which was continuously purged with dry air. The infrared cell was thermostated with a water circulation bath. The resolution was 4 cm^−1^; 256 scans were collected and co-added per sample spectrum, and Fourier-transformed for each sample. Every FTIR spectrum was representative of at least six independent measurements. The infrared spectra of buffer and residual water vapour were subtracted from the infrared spectrum of the sample. Band positions were determined using second derivative spectra. Amide I band of OAD complex and its subunits was fitted using PeakFit software (Scientific Solutions, Switzerland) using the second derivative procedure.

## Supporting Information

Figure S1 Amide I fitted spectra of OAD and OAD subunits: OAD (a), αγ (b), biotinylated α (c), and nonbiotinylated α (d) subunits. Red dotted lines - experimental spectrum; black full lines - fitted curve. Inserted tables contain peak position and proportion of the secondary structures.
(2.06 MB TIF)
