# Supplementary figures and images for: Structure-Function Relations in Oxaloacetate Decarboxylase Complex. Fluorescence and Infrared Approaches to Monitor Oxomalonate and Na+ Binding Effect
Source: PLoS One. 2010 Jun 3;5(6):e10935. doi: 10.1371/journal.pone.0010935 (PMC2881705; doi:10.1371/journal.pone.0010935)

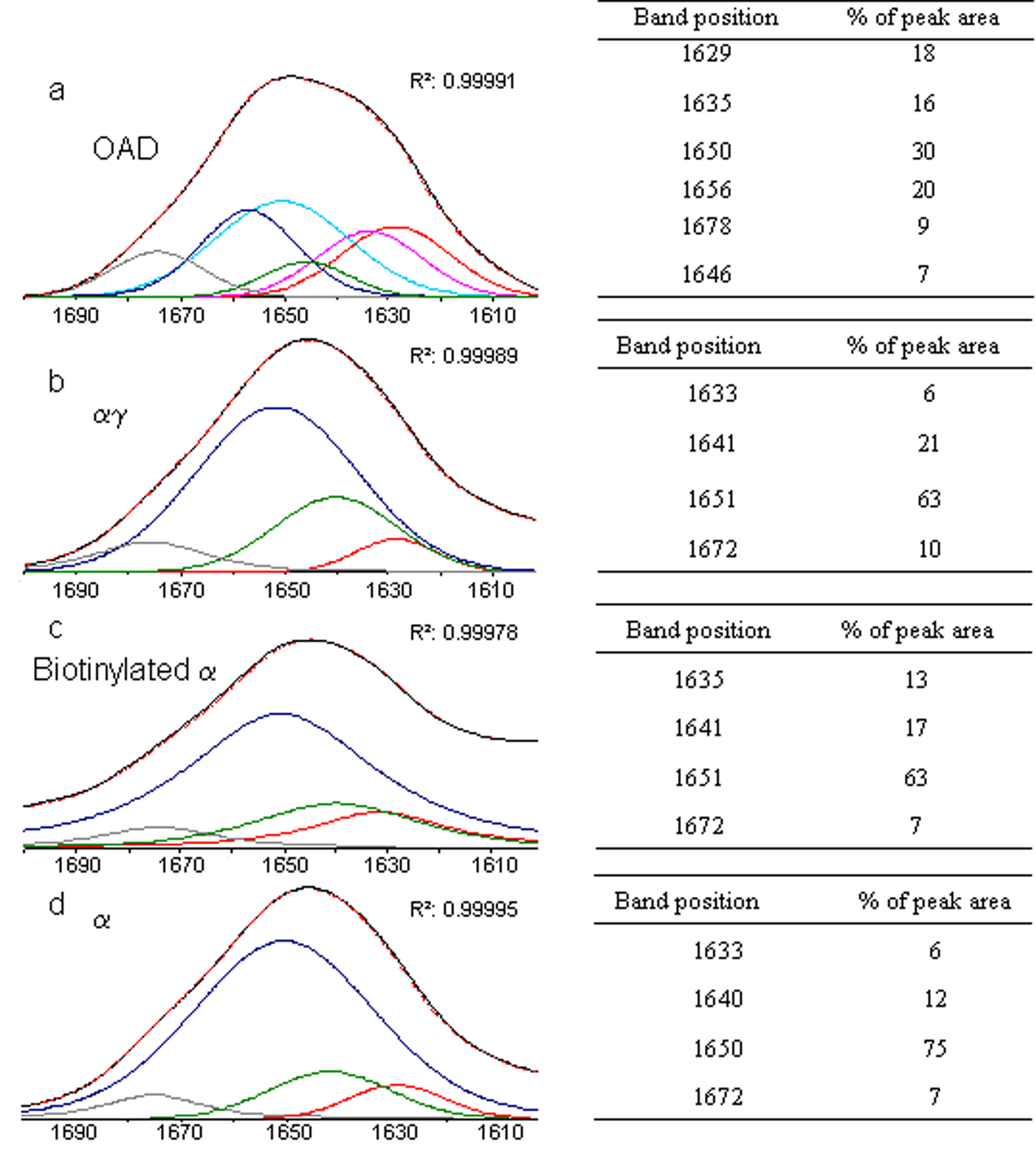

Supplement: Figure S1 — Amide I fitted spectra of OAD and OAD subunits: OAD (a), αγ (b), biotinylated α (c), and nonbiotinylated α (d) subunits. Red dotted lines - experimental spectrum; black full lines - fitted curve. Inserted tables contain peak position and proportion of the secondary structures. (2.06 MB TIF) [file pone.0010935.s001.tif]
